# Retinoblastoma

**DOI:** 10.1186/1750-1172-1-31

**Published:** 2006-08-25

**Authors:** Isabelle Aerts, Livia Lumbroso-Le Rouic, Marion Gauthier-Villars, Hervé Brisse, François Doz, Laurence Desjardins

**Affiliations:** 1 Pediatric Oncology Department, Institut Curie, Paris, France; 2 Ophthalmology Department, Institut Curie, Paris, France; 3 Genetics Department, Institut Curie, Paris, France; 4 Radiology Department, Institut Curie, Paris, France

## Abstract

Retinoblastoma is a rare eye tumor of childhood that arises in the retina. It is the most common intraocular malignancy of infancy and childhood; with an incidence of 1/15,000–20,000 live births. The two most frequent symptoms revealing retinoblastoma are leukocoria and strabismus. Iris rubeosis, hypopyon, hyphema, buphthalmia, orbital cellulites and exophthalmia may also be observed. Sixty per cent of retinoblastomas are unilateral and most of these forms are not hereditary (median age at diagnosis two years). Retinoblastoma is bilateral in 40% of cases (median age at diagnosis one year). All bilateral and multifocal unilateral forms are hereditary. Hereditary retinoblastoma constitutes a cancer predisposition syndrome: a subject constitutionally carrying an *RB1 *gene mutation has a greater than 90% risk of developing retinoblastoma but is also at increased risk of developing other types of cancers. Diagnosis is made by fundoscopy. Ultrasound, magnetic resonance imaging (MRI) and computed tomography (CT) scans may contribute to diagnosis. Management of patients with retinoblastoma must take into account the various aspects of the disease: the visual risk, the possibly hereditary nature of the disease, the life-threatening risk. Enucleation is still often necessary in unilateral disease; the decision for adjuvant treatment is taken according to the histological risk factors. Conservative treatment for at least one eye is possible in most of the bilateral cases. It includes laser alone or combined with chemotherapy, cryotherapy and brachytherapy. The indication for external beam radiotherapy should be restricted to large ocular tumors and diffuse vitreous seeding because of the risk of late effects, including secondary sarcoma. Vital prognosis, related to retinoblastoma alone, is now excellent in patients with unilateral or bilateral forms of retinoblastoma. Long term follow-up and early counseling regarding the risk of second primary tumors and transmission should be offered to retinoblastoma patients.

## Disease name

Retinoblastoma.

## Definition

Retinoblastoma is a rare eye tumor of childhood that arises in the retina and represents the most common intraocular malignancy of infancy and childhood [1]. It may occur at any age but most often it occurs in younger children, usually before the age of two years.

## Epidemiology

The incidence is 1 in 15,000–20,000 live births. In 60% of cases, the disease is unilateral and the median age at diagnosis is two years. Of these cases, 15% are hereditary. Retinoblastoma is bilateral in about 40% of cases with a median age at diagnosis of one year. All bilateral and multifocal unilateral forms are hereditary.

## Clinical description

**Figure 1:**
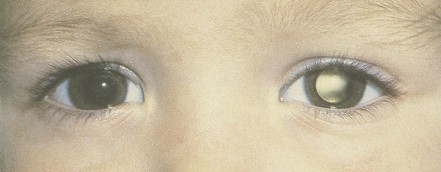
Leukocoria.

Leukocoria (white reflection in the pupil) and strabismus are the most frequent clinical manifestations of retinoblastoma (Figure 1). Leukocoria is initially inconstant, visible only at certain angles and under certain light conditions. This sign may be seen on flash photography. Strabismus, when present, becomes rapidly constant, reflecting impairment of the vision. These signs are still all-too-often overlooked and justify an ophthalmological consultation with ocular fundus examination. Some other signs may be observed, including iris rubeosis, hypopyon, hyphema, buphthalmia, orbital cellulitis, and exophthalmia. Some children with retinoblastoma may have no symptoms. Screening in case of familial history or dysmorphic syndrome with a 13q14 deletion [2] may lead to diagnosis of retinoblastoma. Most affected children are diagnosed before the age of five years.

## Etiology

Retinoblastoma is the first disease for which a genetic etiology of cancer has been described and the first tumor suppressor gene identified. Knudson in 1971 developed the hypothesis that retinoblastoma is a cancer caused by two mutational events [3]. This led to the understanding that there are two forms of retinoblastoma, germinal and non germinal. Loss or mutations of both alleles of the retinoblastoma gene *RB1*, localized to chromosome 13q1.4 [4], are required to develop the disease. In hereditary cases (representing approximately 55% of the cases) patients carry a germline inactivated *RB1 *allele present in all cells in the body and somatic loss of the second allele in retinal cells. Germinal *RB1 *mutations with a high penetrance rate (> 90%) concern all patients with bilateral retinoblastoma as well as 15% of patients with the unilateral form. In non hereditary cases (45% of all patients) both *RB1 *alleles are inactivated somatically in a single developing retinal progenitor cell. In these cases, retinoblastoma is always unilateral and unifocal.

The *RB1 *gene, composed of 27 exons, encodes for a 110 kD nuclear phospoprotein (pRB). pRB, a tumor suppressor gene, is a regulator at the cell cycle check point between G1 and entry into S phase. In the knock-out mouse model of retinoblastoma, Zhang *et al*. demonstrated that pRB is required for appropriate exit from the cell cycle of retinal progenitor cells and for rod development [5]. Numerous studies indicate that other molecular events, in addition to the loss of pRB, are necessary for tumorigenesis (chromosomal gain +1q, +6p; chromosomal loss -16, -16q, -17, -17p) [6,7].

A novel theory about the potential mechanism of retinoblastoma tumor development was proposed in response to the observation that spontaneous unilateral retinoblastoma may be more frequent in non industrialized countries. Orjuela *et al*., in a study evaluating the presence of oncogenic human papilloma virus (HPV), concluded that pRB inactivation could be caused in part by the oncoprotein HPV E7 [8]. Environment (low folate intake during pregnancy) was also postulated to play a role in the risk of retinoblastoma occurrence because of the increased incidence of unilateral retinoblastoma in developing countries [9].

## Diagnostic methods

**Figure 2:**
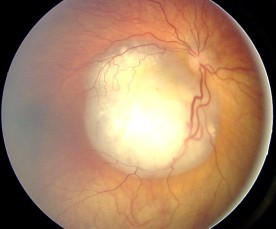
Ocular fundus aspect of retinoblastoma.

An examination of the ocular fundus under general anesthesia leads to diagnosis. The lesion appears as a white tumor with angiomatous dilatation of the vessels (Figure 2).

**Figure 3:**
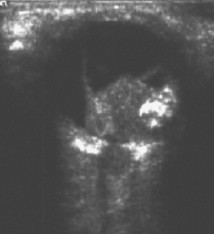
Ultrasound of retinoblastoma.

Ocular ultrasound demonstrates a mass more echogenic than the vitreous, with fine calcifications. Retinal detachment may also be observed in exophytic forms (Figure 3).

**Figure 4:**
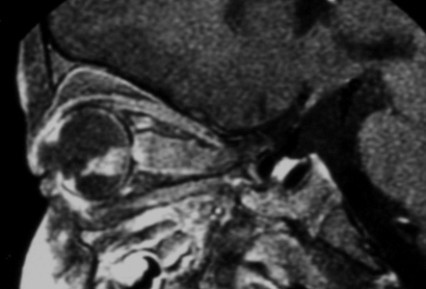
MRI pattern of retinoblastoma with optic nerve involvement (sagittal enhanced T1-weighted sequence).

**Figure 5:**
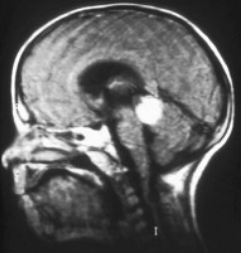
Aspect of trilateral retinoblastoma (MRI).

Magnetic resonance imaging (MRI) is the imaging modality of choice to assess the local extension. The mass has a signal equivalent to or a slightly more intense than that of the vitreous on T1-weighted sequences, with a relatively low-intensity signal on T2-weighted sequences (Figure 4). Fine calcifications are not visible. MRI can depict extensions to the optic nerve, anterior chamber and orbital fat. MRI may be useful for distinguishing retinoblastoma from pseudotumoral conditions such as Coats disease or eye malformations and to diagnose rare cases of trilateral retinoblastoma (third tumor in the pineal gland or parasellar region) (Figure 5) [10].

Computed tomography (CT) typically shows an intraocular mass with a higher density than the vitreous body, calcified in 90% of cases and moderately enhanced after iodine contrast agent injection.

## Differential diagnosis

The rare, invasive, diffuse forms of retinoblastoma, usually not calcified, are very difficult to diagnose due to their atypical and radiological features, mimicking an advanced form of Coats disease and *Toxocara Canis *eye infection [10]. Coats disease corresponds to a unilateral retinal disease with telangiectasia and subretinal exudates. Persistence of the primitive vitreous may also be mistaken for retinoblastoma [11].

## Genetic counseling

Genetic counseling should be proposed to every parent having a child with retinoblastoma and to patients having a familial history of retinoblastoma. During this consultation, the progress of management of retinoblastoma and anticipated examination schedule for testing should be presented to the family. An analysis of the child's personal and familial history, and direct and indirect molecular studies should be performed by the geneticist.

Young age at diagnosis, multifocal nature of the tumor, presence of psychomotor retardation and a malformative syndrome, and presence of a familial history of retinoblastoma and retinoma suggest a genetic predisposition for the disease. The risk of transmission is a function of the familial history and the type of retinoblastoma. In case of hereditary retinoblastoma, the risk of transmission is 50%. In case of unilateral, unifocal, non familial retinoblastoma, the risk of transmission is 5%.

Genetic analysis in affected children may include the following molecular tests:

• *Direct search for a constitutional mutation of the RB1 gene performed on the constitutional DNA*. The mutation detection rate is very high in hereditary forms [12]. No preferential mutation or "hot spot" has been identified in the *RB1 *gene [13-16].

• *Indirect demonstration of the allele carrying the mutation in cases of familial history*. This test consists of identifying intragenic or *RB1 *flanking markers common to all affected members of the family.

• *Tumor loss of heterozygosity evaluation*. This technique requires tumor material and allows determination of which allele is remaining and carrying the mutation.

During this consultation, patients should be informed of the risks of transmission and of second primary tumor development [17,18]. At present, there is no evidence that a particular abnormality of the *RB1 *gene is associated with a higher risk of second cancer.

## Antenatal diagnosis

Antenal diagnosis may be proposed in case of hereditary form with an identified *RB1 *mutation. Indirect detection of the allele carrying the mutation may be used.

Several groups have started to investigate the use of preimplantation genetic diagnosis (PGD) for families who have children with germinal retinoblastoma. Abramson *et al*. recently reported the first live birth of a healthy newborn to a couple in which the father had an history of germinal retinoblastoma [19].

## Management including treatment

Treatment of retinoblastoma is based on the bilateral or unilateral characteristics of the retinoblastoma, on the staging performed by the fundoscopy and the extension of the disease.

### Work-up for retinoblastoma

The fundus is examined under general anesthesia by indirect ophthalmoscopy allowing visualization of the entire retina, from the posterior pole to the most anterior zone. It determines:

• the unilateral or bilateral nature of the lesions

• the number of tumors

• their situation in the retina (posterior pole, anterior retina)

• the tumor size (diameter, thickness)

• the subretinal fluid and tumor seeds

• the vitreous seeding: localized or diffuse

• the anatomical relations with the optic disc and macula

All these parameters should be taken into account for grouping the retinoblastoma and for making therapeutic decisions.

Two classifications are currently used for grouping intraocular retinoblastoma: 1) the Reese Ellsworth classification, according to the chance of preserving the eye using external beam radiotherapy (Table 1 and 2) the new ABC classification, according to the chance of preserving the eye using all modern therapeutic approaches (Table 2) [20]. An international retinoblastoma classification (Table 3) covering the whole spectrum of the disease (from intraretinal to the presence of overt extra-ocular extension) has been recently proposed [21]. In addition, a proposal for a substaging (according to the histopathological features of enucleated specimens) may further help to differentiate patients with intraocular disease. The intraorbital staging is based on CT or MRI imaging, which must be performed in almost all patients. Radiologically visible extension to the retrolaminar optic nerve must be investigated, especially in case of optic disc involvement, as it determines the surgical approach for enucleation. Distant staging looking for metastasis may also be performed when enucleation is necessary and histopathological risk factors have been identified (lumbar puncture, bone marrow aspiration) [22] (Table 4).

**Table 1:** The Reese Ellworth classification

**Group I**
a) solitary tumor, < 4 disc diameters in size, at or behind the equator
b) multiple tumor, none > 4 disc diameters in size, at or behind the equator
**Group II**
a) solitary tumor, 4–10 disc diameters in size, at or behind the equator
b) multiple tumor, 4–10 disc diameters in size, behind the equator

**Group III**
a) any lesion anterior to the equator
b) solitary tumor >10 disc diameters behind the equator

**Group IV**
a) multiple tumors, some >10 disk diameters
b) any lesion extending anteriorly to the ora serrata

**Group V**
a) massive tumors involving more than half the retina
b) vitreous seeding

**Table 2:** The ABC classification

**Group A: **small tumors away from foveola and disc
• Tumors <3 mm in greatest dimension confined to the retina and
• Located at least 3 mm from the foveola and 1.5 mm from the optic disc
**Group B: **all remaining tumors confined to the retina
• All other tumors confined to the retina and not in group A
• Subretinal fluid (without subretinal seeding) < 3 mm from the base of the tumor

**Group C: **local subretinal fluid or vitreous seeding
• Subretinal fluid alone >3 mm and < 6 mm from the tumor
• Vitreous or subretinal seeding < 3 mm from the tumor

**Group D: **diffuse subretinal fluid or seeding
• Subretinal fluid > 6 mm from the tumor
• Vitreous or subretinal seeding > 3 mm from the tumor

**Group E: **presence of any one or more of these poor prognosis features
• More than 2/3 of the globe filled with tumor
• Tumor in the anterior segment or anterior to the vitreous
• Tumor in or on the ciliary body
• Iris neovascularisation
• Neovascular glaucoma
• Opaque media from hemorrhage
• Tumor necrosis with aseptic orbital celullitis
• Phthisis bulbi

**Table 3:** International retinoblastoma classification

**Stage 0: **Patients treated conservatively (subject to presurgical ophthalmologic classifications)
**Stage I: **Eye enucleated, completely resected histologically

**Stage II: **Eye enucleated, microscopic residual tumor

**Stage III: **Regional extensiona
a) Overt orbital disease
b) Preauricular or cervical lymph node extension

**Stage IV: **Metastatic disease
a) Hematogenous metastasis:
1. single lesion
2. multiple lesions
b) CNS extension:
1. Prechiasmatic lesion
2. CNS mass
3. Leptomeningeal disease

**Table 4:** Staging of retinoblastoma

Ocular fundus under general anesthesia +	→	Any patient with retinoblastoma Schema, photographs, ultrasonography Reese grouping, new grouping
Brain and orbit CT scan or MRI +	→	Almost any patient with retinoblastoma (except neonatal screened patients with tumor respecting the head of optic nerve)
CSF cytology Bone marrow cytohistology +	→	When enucleation is necessary and shows histopathologic risk factors
Brain and spinal axis MRI Bone scan	→	Only in case of orbital, lymph node and/or distant metastatic diseases

CT – computed tomography

MRI – magnetic resonance imaging

CSF – cerebrospinal fluid

### Management of localized form of retinoblastoma

Treatment approaches have changed over the past decade. Many centers in recent years tried to increase the use of chemotherapy and focal treatment methods, such as transpupillary thermotherapy, photocoagulation and cryotherapy. These treatment methods were employed in an effort to avoid the use of external beam radiation therapy. Intraocular retinoblastoma continues to be managed by a large number of treatment modalities including: enucleation, transpupillary thermotherapy, cryotherapy, laser photocoagulation, brachytherapy, external beam radiotherapy and chemotherapy (systemic and local delivery).

### Management of extensive unilateral retinoblastoma

**Figure 6:**
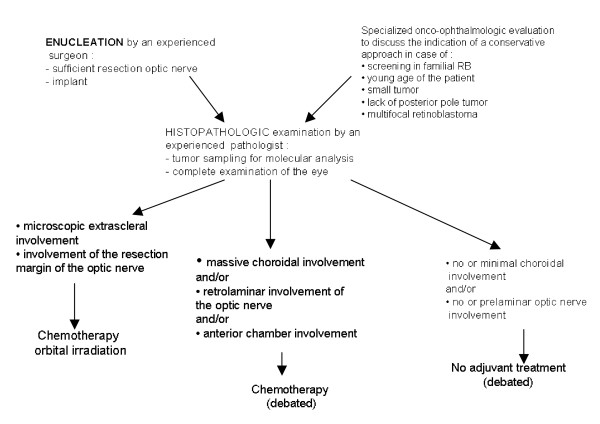
Management of extensive unilateral retinoblastoma.

Enucleation is still often performed for extensive unilateral retinoblastoma because of the elevated frequency of extensive tumors (Group V, Reese Ellsworth classification for retinoblastoma). The role of adjuvant treatment (that aims to decrease the extraocular relapse) is debated; experienced pathologists advise avoiding this toxic treatment in the majority of patients who would not benefit from adjuvant chemotherapy [23-27]. Combinations of different agents are used, such as carboplatin-etoposide and vincristine-cyclophosphamide-doxorubicin. Orbital irradiation, external beam radiotherapy and interstitial radiotherapy [28] is performed in the case of microscopically incomplete resection. The management of extensive unilateral retinoblastoma is summarized in the following arborescence (Figure 6).

### Management of bilateral retinoblastoma

**Figure 7:**
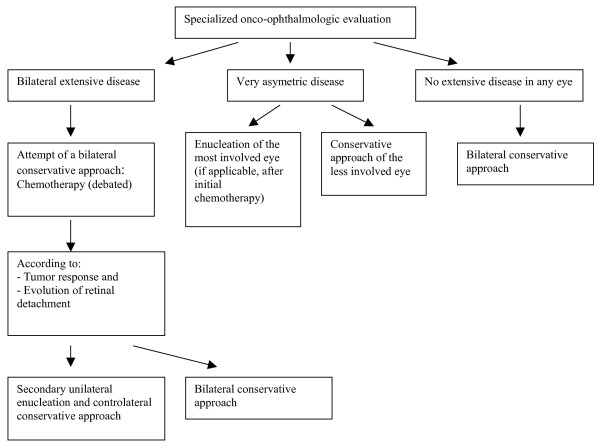
Management of bilateral retinoblastoma.

In case of bilateral retinoblastoma, some conservative approaches for treatment of at least one eye have been developed (Figure 7). Their application depends on the number of tumors, their situation in relation to the macula and the optic disc, the presence of partial or total retinal detachment, the presence of invasion of the vitreous and preretinal space, the age at diagnosis and the presence of a familial history.

The first conservative treatment used in the majority of patients was external beam radiotherapy. Its use has declined in many centers because numerous studies have demonstrated serious adverse effects including cosmetic, opthalmological, endocrine and neuro-cognitive sequelae, and, in particular, the increased risk of a second non-ocular cancer in survivors of germinal retinoblastoma [17,18]. Some of these adverse effects and the risk of development of a second cancer may be more frequent if external beam radiotherapy is delivered before one year of age [29]. However, this age-limit for increased risk of a second cancer is not constantly observed: in our own series of secondary sarcomas within the radiation field, 8/21 patients were irradiated after one year of age [17]. External beam radiotherapy remains an excellent way of preserving eye vision in certain clinical situations (massive intraocular tumor, diffuse vitreous seeding).

Some other conservative techniques have been developed: cryotherapy [30], brachytherapy [31], transpupillary thermochemotherapy [32,33], systemic or local chemotherapy with or without focal laser. Their indication depends on the number, the localization (anterior retina or posterior pole) and the size of the tumors.

Neoadjuvant chemotherapy may be administered in order to make ocular tumors accessible to conservative management, to allow conservative management other than external beam radiation, to improve the visual prognosis by promoting retinal reapplication and by decreasing or eliminating macular invasion, and to allow creation of healthy retinal space between the tumor and the optic disc. Cytotoxic agents used as a combination of two or three drugs are carboplatin, etoposide and vincristine [32,34-39]. The choice of agents, as well as the number and frequency of cycles, may vary. In cases of paramacular tumors, chemotherapy alone may be used [35].

After neoadjuvant chemotherapy, local treatment is used in most of the cases. It includes laser treatment alone and thermochemotherapy for tumors of the posterior pole, and cryotherapy and brachytherapy for tumors of the anterior pole. In case of vitreous seeding, external beam radiotherapy, protontherapy and local administration of carboplatin by subconjunctival injection are indicated [40].

### Follow-up for bilateral retinoblastoma

Fundoscopy, under general anesthesia until the age of four or five years, should be performed every month during the first year after the end of treatment. The interval between examinations may then be gradually increased to one examination every three months, even in case of unilateral retinoblastoma (because of the risk of late bilateral involvement) [41]. The objective is to detect new tumors and ocular complications related to treatment. MRI and ultrasound are necessary in case of vitreous hemorrhage due to rupture of vitreoretinal adhesions or cataract, in order to detect tumor progression before performing surgery of these complications. Assessment of the orbital cavity after enucleation should also be performed.

MRI could be useful to detect trilateral retinoblastoma. Some argue that the infrequency of trilateral retinoblastoma does not warrant routine neuroimaging in patients in whom ocular disease is diagnosed [42]. However, Blach and Col reported that patients with bilateral retinoblastoma have 6 to 8% risk of developing trilateral retinoblastoma until the age of six years and that early detection (by MRI) in asymptomatic patients gives a better chance for a cure [43]. Prognosis of patients with trilateral retinoblastoma remains poor, with most patients dying from progressive disease within 2 years of diagnosis. Meta-analysis of trilateral retinoblastoma found that trilateral retinoblastoma was detected earlier in patients undergoing routine serial imaging; in addition these patients survived longer than those who did not undergo such imaging [44]. Suggested screening programs for detection of trilateral retinoblastoma widely vary [45]. Thus, interpretation of a pineal tumor in a patient with bilateral retinoblastoma must be carefully done, taking into consideration the recently described strong relationship between retinoblastoma and pineal cysts [46]. Pineal cysts may represent a variant of trilateral retinoblastoma and warrant a less aggressive therapeutic approach.

Ophthalmologic follow-up should include a complete assessment of visual function (visual acuity, refraction disorders) [47]. Pediatric follow-up is useful for detecting sequelae of the treatment (second tumors, platin secondary ototoxicity) and for estimating the visual handicap.

### Management of retinoblastomas with extraocular involvement

Extraocular involvement of retinoblastoma may concern the soft tissues of the orbit, pretragal and cervical lymph nodes, and metastases affecting bone, bone marrow and the central nervous system (CNS). These forms, becoming rare in industrialized countries, are still frequent in developing countries. Intensification of chemotherapy, based on the use of high dose carboplatin, has greatly improved the prognosis of the extraocular forms of retinoblastoma, except in cases of CNS involvement [26,48,49].

## Prognosis

Vital prognosis, related to retinoblastoma alone, is now excellent in patients with unilateral or bilateral forms of retinoblastoma, with a cure rate of 95% in industrialized countries. The preservation of visual function depends on ocular preservation, the initial tumor volume, the anatomical relationships of the tumors with the macula and the optic disk and the adverse effects of the treatments (cataract, vitreous hemorrhage). Long term survival in the hereditary form is threatened by the risk of occurrence of second tumors [17,50]. A recently published study reported a cumulative incidence for developing a new cancer at 50 years after diagnosis of retinoblastoma of 36% for hereditary and 5.7% for non hereditary patients [18]. Patients treated for hereditary retinoblastoma are at an increased risk of developing non-ocular malignancies due to a mutation in the second *RB1 *allele in different tissues. External beam radiation, when administered before the first year of life, and chemotherapy may also increase the risk of development of second neoplasms [29,51]. The most frequent tumors encountered are osteogenic sarcomas of the skull and long bones, soft tissue sarcomas, cutaneous melanomas, brain tumors, and lung and breast cancer. Although external beam radiotherapy is less widely used than in the past, the risk of development of a second tumor is still significant and patients should be informed and carefully followed-up [52].

## Unresolved questions

Despite the identification of the *RB1 *gene and the current insight into the function of pRB, the understanding of the sequence that leads to human retinoblastoma is still incomplete. Recent development of new animal models of retinoblastoma will increase the knowledge on tumorigenesis and provide an opportunity to develop treatment strategies [5]. Although retinoblastoma has a good prognosis in industrialized countries, mortality due to development of a second tumor remains high. Development of a non-mutagenic therapy (such as photodynamic therapy) could be interesting, particularly in case of hereditary retinoblastoma [53-55]. Gene therapy for treatment of retinoblastoma is under evaluation [56].

In developing countries, retinoblastoma is unfortunately accompanied by a high mortality rate due to a significantly delayed diagnosis made at advanced stages of the disease.
